# Vitamin D Deficiency as a Predictive Factor of Transient Hypocalcemia after Total Thyroidectomy

**DOI:** 10.1155/2020/8875257

**Published:** 2020-10-10

**Authors:** A. Bove, C. Dei Rocini, R. M. Di Renzo, M. Farrukh, G. Palone, S. Chiarini, T. Staniscia

**Affiliations:** ^1^Department of Medicine, Dentistry and Biotechnology, University “G. D'Annunzio”, Via dei Vestini, 66100 Chieti, Italy; ^2^Department of Medicine and Ageing Sciences, University “G. D'Annunzio”, Chieti-Pescara, Italy

## Abstract

**Background:**

Total thyroidectomy (TT) is recommended in the treatment of malignant and benignant thyroid diseases, and, to date, transient hypocalcemia is the most frequent complication after the procedure. We prospectively evaluated the role of vitamin D deficiency as a predictor of postoperative hypocalcemia.

**Methods:**

This is a prospective cohort study which was conducted between January 2016 and April 2019. A total of 177 consecutive patients (141 (79.7%) women and 36 (20.3%) men) who underwent TT were included in the current study. Hypocalcemia occurred when serum calcium levels were below 8.0 mg/dL or 1.10 mmol/L. Patients were divided into two groups (Group 1, normocalcemic; Group 2, hypocalcemic) and were assessed taking into consideration preoperative serum 25-hydroxy vitamin D (25-OHD) levels, preoperative serum calcium levels, thyroid hormone levels, sex, body mass index (BMI), and smoking habits. Vitamin D deficiency was defined as 25-OHD levels <25 ng/mL.

**Results:**

The incidence of postoperative asymptomatic and symptomatic hypocalcemia in the two groups was 19.8% and 15.8%, respectively. Preoperative 25-OHD level was significantly different between Group 1 and Group 2 (31.5 ± 15.0 ng/mL vs 18.7 ± 9.8 ng/mL,*p*=0.017). Logistic regression analysis revealed that preoperative vitamin D deficiency was a significant predictive factor of postoperative hypocalcemia (*p*=0.012), and, specifically, the risk of hypocalcemia increased 15-fold in patients with a preoperative vitamin D level <25 ng/mL (odds ratio [OR], 14.8).

**Conclusions:**

Postoperative hypocalcemia is significantly associated with low preoperative levels of serum 25-OHD. Our studies demonstrate that vitamin D deficiency (<25 ng/mL) is an independent predictive factor of postoperative hypocalcemia.

## 1. Introduction

Total thyroidectomy (TT) is a procedure that is used to treat malignant and benign thyroid diseases [1].

In order to achieve optimal results, a thorough knowledge of the anatomical structures, including the identification of the laryngeal nerves and parathyroids, is mandatory [2].

According to the literature data, even after performing an optimal surgical technique, transient hypocalcemia is still possible and remains the most frequent complication after TT, with an incidence range varying from 1.6%–7% to 38–53% [3]. This high discrepancy in the data is due to the absence of an established definition of transient postoperative hypocalcemia [4].

The etiology of hypocalcemia is multifactorial and includes posttraumatic stupor of parathyroid glands, incidental parathyroidectomy, surgeon's expertise, extent of surgical procedure, and hyperthyroidism [5].

Hypocalcemia has implications for both patients' health and for healthcare expenses that increase due to a longer postoperative hospital stay.

Furthermore, on the matter of calcemia and calcium homeostasis, vitamin D has a significant role. Vitamin D can be acquired through food or supplements or through exposure to ultraviolet light that leads to its synthesis in the layers of the skin. Subsequently, it needs to be converted to 25-hydroxyvitamin D (25-OHD) by the liver, and serum 25-OHD level is the parameter used to detect the overall status of vitamin D in the body. A further hydroxylation of 25-OHD in the kidney leads to the most active form of vitamin D known as 1,25-dihydroxyvitamin D (1,25-(OH)2D).

Several studies have tried to use the serum vitamin D level as a preoperative marker of postoperative hypocalcemia, but the results in the literature are not conclusive [6, 7].

The aim of our study is to investigate and elucidate the controversial role of 25-OHD deficiency as a predictive factor of transient postoperative hypocalcemia in patients undergoing TT.

## 2. Method

From January 2016 to April 2019, a total of 177 patients (36 (20.3%) men and 141 (79.7%) women) underwent TT. The mean age of the patients was 51.4 ± 13.8 years. The female/male ratio was 3.9/1 (*n* = 141/36).

Each TT was performed by two experienced and skilled surgeons using the vascular resection technique, with the identification and isolation of the inferior laryngeal nerves and at least 3 parathyroid glands remaining in situ. The energy-based device was used occasionally.

Patients undergoing reoperation and/or lymphadenectomy were excluded. Patients either affected by other diseases or using drugs that could interfere with the metabolism of calcium were also excluded.

The serum calcium level was controlled on the first and the second postoperative days.

Hypocalcemia was defined as serum calcium levels either below 8.0 mg/dL or 1.10 mmol/L, thus requiring proper replacement therapy. Patients were divided into two groups: Group 1—normocalcemic and Group 2—hypocalcemic, and were assessed taking into consideration preoperative serum 25-OHD levels, preoperative serum calcium levels, thyroid hormone levels, sex, body mass index (BMI), smoking habits, and operative time.

Vitamin D deficiency was defined as serum 25-OHD levels <25 ng/mL.

The data of < than 25 ng/ml have been defined as the reference value of the vitamin D deficiency using the average between the different accepted guidelines (cutoff: 20 or 30 ng/ml).

### 2.1. Statistical Analysis

The continuous variables were summarized as mean and standard deviation (SD) or median and interquartile range (IQR), according to their distribution. Qualitative variables were summarized as frequencies and percentages. The Shapiro–Wilk test was performed to evaluate the distribution of continuous variables. Pearson's chi-square test was performed to compare qualitative variables.

The Mann–Whitney *U* test was performed to compare the continuous variables between normocalcemic and hypocalcemic patients. Univariate and multivariate logistic regression analysis was performed to estimate the risk of hypocalcemia, expressed as odds ratio (OR) and 95% confidence interval (95% CI). Significance was evaluated for *p* < 0.05. The statistical analysis was performed using the IBM SPSS Statistics v.20 software.

## 3. Results

Throughout our study, no intraoperative complications and no mortality occurred. The incidence of transient dysphonia was 0.56% (1/177). After histopathologic evaluation, thyroid cancer was detected in 39 patients (22%) (7 incidental microcarcinomas). After TT, patients were divided into two groups according to serum calcium levels: Group 1, normocalcemic (*n* = 114) (64.4%) and Group 2, hypocalcemic (*n* = 63) (35.6%).

The incidence of postoperative asymptomatic hypocalcemia was 19.8% (35 patients) and that of symptomatic hypocalcemia was 15.8% (28 patients). The symptoms just reported by the patients were paraesthesia.

Group 1 and Group 2 patients were comparable for preoperative parameters such as age, BMI, and smoking habits, while there were significant differences in gender distribution between the two groups, as shown in Table 1 (*x*_2_, *p*=0.044).

There were no significant differences among thyroid pathologies and thyroid functionality, nor on the hypocalcemia rate (*p* > 0.05 in all cases) between the 2 groups (Table 2). There were only small postoperative increases in hospital stay in Group 2 (2.9 ± 1.1 compared to 2.6 ± 0.9) but with no statistical significance (*p* > 0.05) (Table 2). Even the operative time of the two groups does not show considerable differences.

Regarding the preoperative biochemical parameters considered in the study, the results are shown in Table 3. There was no significant difference in preoperative serum albumin, parathormone (PTH), or calcium levels between Groups 1 and 2 (*p* > 0.05); in fact, each patient had a normal preoperative serum calcium level. Conversely, the preoperative serum 25-OHD level was significantly lower for Group 2 patients than for Group 1 patients (18.7 ± 9.8 ng/ml vs 31.5 ± 15.0 ng/ml) (Table 3).

Logistic regression analysis revealed that preoperative vitamin D deficiency was an independent significant variable in the development of hypocalcemia after TT (*p*=0.012) (Table 4). Furthermore, the risk of postoperative hypocalcemia was 15 times greater in those patients with preoperative 25-OHD <25 ng/ml (odds ratio [OR] 14.8, 95% CI). Female gender, obesity, and smoking habits increased the risk of hypocalcemia by 5, 3, and 3.7 times, respectively, although the results were not statistically significant (*p* > 0.05) (Table 4).

A second regression analysis was performed to evaluate the risk of postoperative symptomatic hypocalcemia. Vitamin D deficiency was again a significant independent predictor (*p*=0.045); the risk of postoperative symptomatic hypocalcemia increased 18 times in those patients with preoperative vitamin D <25 ng/ml (OR 18.1, 95% CI).

At a six-month checkup, in 3 patients (2%), the hypocalcemia turned out to be definitive.

## 4. Discussion

Transient hypocalcemia remains the most frequent complication after TT with an incidence ranging from 1.6% to 53% [8]. However, there is no overall, agreed-upon definition of postthyroidectomy hypocalcemia [9]. We have established hypocalcemia as a serum calcium level either below 8.0 mg/dL or below 1.10 mmol/L.

Several studies have investigated predictive factors of hypocalcemia after TT in order to anticipate therapy so as to reduce symptoms and guarantee a timely and safe discharge [10, 11].

For instance, many studies have targeted the dosage of preoperative PTH, intraoperative PTH, and postoperative PTH. These studies proved that PTH is a reliable indicator for the early detection of hypocalcemia [12, 13]. However, the preoperative PTH is usually within a normal range, so the PTH can be considered an early diagnosis indicator rather than a substantial predictive factor.

The aim of our study is to assess, preoperatively, the risk of postoperative low serum calcium levels with the significant advantage of avoiding hypovitaminosis related to hypocalcemia by correcting any vitamin D deficiency. Interest in vitamin D is due to an increasingly widespread occurrence of hypovitaminosis D in the general population to the point where Hollick et al. defined the problem of vitamin D deficiency as a world health issue [14].

Calcium homeostasis is closely related to vitamin D together with PTH. It has been hypothesized that patients with vitamin D deficiency undergoing transient hypoparathyroidism circumstances, such as iatrogenic surgical trauma to the parathyroid glands, are at higher risk of developing hypocalcemia. This is due to the loss of the PTH compensatory mechanism in maintaining normal calcemia. In fact, calcium level regulation in patients with vitamin D deficiency depends on PTH levels. In case of damage to the parathyroid glands, for instance, due to surgical manipulation, a reduction in PTH secretion is induced with a consequent sudden decrease of serum calcium levels in patients with vitamin D deficiency.

Different results have been produced from the numerous studies without reaching a consensus on the role of vitamin D deficiency as a risk factor for transient hypocalcemia after TT.

Griffin et al. [15], in their study, have shown that there is no significant difference in the incidence rate of postthyroidectomy hypocalcemia among different vitamin D groups. However, patients undergoing central neck dissection were included in the study, thus possibly affecting the data and statistical analysis.

A study conducted by Lin et al. [16] on 152 patients who underwent subtotal thyroidectomy showed that vitamin D levels are not predictive of transient hypocalcemia. However, this result could be disputed because in all patients, a postoperative therapy based on calcium carbonate and cholecalciferol was routinely prescribed.

Moreover, from the study of Chia et al. [17], conducted on 103 patients, there is no association between preoperative vitamin D levels and postoperative hypocalcemia although some patients included in the study had parathyroid adenoma or parathyroid hyperplasia.

Likewise, other studies have reported the same negative results [18, 19], but many others have instead confirmed the predictive role of vitamin D deficiency in the onset of postoperative hypocalcemia [20, 21].

Notably, the study conducted by Erbil et al. [22] on 200 patients who underwent total or subtotal thyroidectomy reported that vitamin D levels below 15 ng/mL were associated with a significant increase of postoperative hypocalcemia risk.

In addition, in another study by Erbil et al. [23] concerning 130 patients who underwent TT for nontoxic multinodular goiter, the vitamin D deficiency (cutoff: 25 ng/mL) appears to be a significant risk factor for hypocalcemia (an increase in risk of 15 times). Moreover, from this study, it emerged that preoperative vitamin D deficiency is more significant than a low PTH value in predicting postsurgical hypocalcemia.

Kirkby-Bott et al. [24], in their study on 166 patients, also demonstrated a significant association between vitamin D levels below 25 ng/mL and postoperative hypocalcemia. In addition, they demonstrated that preoperative vitamin D values >50 ng/mL were valuable protective factors for transient hypocalcemia.

In our study, we found that serum preoperative vitamin D levels were significantly lower in the group of patients who developed hypocalcemia (19.57 (7.10–24.84) ng/mL) compared to normocalcemic patients (28.7 (23.5–40.5) ng/mL) (*p*=0.012).

Vitamin D deficiency is defined as serum 25-OHD level <25 ng/mL (as in the studies of Erbil et al. [23] and Kirkby-Bott et al. [24]).

Our logistic regression analysis showed an increase in transient hypocalcemia after TT in patients with vitamin D deficiency. Furthermore, it is now possible to quantify the increased risk due to the preoperative vitamin D deficiency as, both in our work and in the available literature, this is repeatedly a 15-fold increase compared to the population with 25-OHD within the normal range (25-OHD >25 ng/ml).

Our results corroborate the initial hypothesis of hypovitaminosis D as an independent predictor of transient hypocalcemia after TT, and they are comparable with the studies described above. Given the significance of the statistics obtained (*p* < 0.05), these results may represent a valid contribution to the controversy in the international literature.

According to our results, it is possible to state the importance of vitamin D in the regulation of calcium homeostasis and, above all, its role in compensating PTH reductions, as occurs in the case of thyroid surgery. In fact, in patients with sufficiently high vitamin D levels (25-OHD >25 ng/mL), hypocalcemia appears with a much lower frequency than in patients with insufficient serum concentrations.

As concerns the recognition of parathyroids glands, despite what is reported by some authors [9], no significant differences were obtained between the two study groups. This is in accordance with the study of Erbil et al. which states that the clinical significance of accidental parathyroidectomy is not well defined, and the matter of how many parathyroids to spare in order to obtain normal serum calcium level still remains unsolved [22].

This difference in opinion in the literature arises firstly because the identification of the parathyroids is purely subjective; furthermore, following a correct identification and a correct preservation attempt, one is never certain to have perfectly preserved the gland and its vascular supply. Therefore, even after performing what is believed to be correct preservation of parathyroids, there still is the possibility of damage causing a decrease in secretion, thus leading to hypocalcemia.

A relevant issue to ponder is the incidence of asymptomatic hypocalcemia, which reaches up to 19.8% of cases in our experience. In this particular situation, there is no agreement as to the need for therapy, and indeed, many discard this option [25]. Our logistic regression analysis showed that preoperative vitamin D deficiency is also a significant predictive factor for symptomatic calcemia. These data could be helpful in assessing the decision of not treating patients with low levels of serum calcium and preoperative levels of vitamin D within the normal range.

Other significant data that emerged from our study concern the distribution of women in the two groups. There is a significant increase in the percentage of women who suffered from hypocalcemia compared to those remaining normocalcemic (*p* < 0.05). Our logistic regression analysis showed that female sex increased 5 times the risk of postoperative hypocalcemia, although the result was not statistically significant (*p* > 0.05) (Table 4). However, these data are supported in the literature by other studies [26, 27] in which the female gender is a significant risk factor for postthyroidectomy hypocalcemia.

This study does not show a significant difference in the incidence of postoperative hypocalcemia among patients with hyperfunctioning and those with normal functioning thyroid disease, such as in the study of Kirkby-Bott et al. [24], but it shows that neoplastic pathology does play a role in the risk of hypocalcemia even though it is not substantial (*p* > 0.05) (Table 4).

Although not statistically significant (*p* > 0.05), obesity (BMI values above 30 kg/m^2^) and smoking habits were found to be factors that increased the risk of postoperative hypocalcemia by 1.8-fold and 3.7-fold, respectively.

## 5. Conclusions

In conclusion, postoperative hypocalcemia is significantly associated with low preoperative levels of serum 25-OHD. This study demonstrates that vitamin D deficiency (25-OHD <25 ng/mL) is an independent predictive factor of transient hypocalcemia following TT.

In line with our study, and with the aim to reduce the incidence of postoperative hypocalcemia, we recommend assessing the level of vitamin D about 1–2 months before thyroid surgery and eventually prescribing adequate doses of cholecalciferol or 25-hydroxycholecalciferol for patients with levels lower than 25 ng/mL.

Achieving physiological levels of vitamin D allows patients to compensate any reduction of PTH that may occur after TT.

We thus conclude that, according to our results, an early recognition and prevention of hypocalcemia can be achieved by a preoperative vitamin D assessment as a predictive factor of occurrence of transient hypocalcemia after TT.

## Figures and Tables

**Table 1:** Patients' characteristics.

	Normocalcemic	Hypocalcemic	*p* value
Patient number, *n* (%)	114 (64.4)	63 (35.6)	—

Age, mean ± SD	52.6 ± 14.0	50.1 ± 13.6	0.172^a^

Gender, *n* (%)	—	—	**0.044** ^b^
Female	86 (75.4)	55 (87.3)	—
Male	28 (24.6)	8 (12.7)	—

Body mass index, mean ± SD	27.5 ± 5.2	26.5 ± 6.4	0.072^a^
Underweight	2 (1.8)	1 (1.6)	0.147^b^
Normal weight	34 (29.8)	28 (44.4)	—
Overweight/obese	78 (68.4)	34 (54.0)	—

Menopause, *n* (%)	50 (43.9)	23 (36.5)	0.061^b^

Smoking, *n* (%)	30 (26.3)	15 (23.8)	0.946^b^

^a^Mann–Whitney *U* test. ^b^Pearson's chi-square test.

**Table 2:** Underlying disease, thyroid functional status, and operative and discharge times.

	Normocalcemic	Hypocalcemic	*p* value^a^
*Underlying disease n (%)*			
Multinodular thyroid disease	84 (72.0)	51 (81.0)	0.207
Cervico-mediastinal goiter	16 (14.0)	9 (14.3)	0.999
Graves' disease	7 (6.1)	4 (6.3)	0.999
Hashimoto's thyroiditis	8 (7.0)	2 (3.2)	0.498
Hurthle adenoma	3 (2.6)	2 (3.5)	0.999
Follicular adenoma	1 (0.9)	2 (3.2)	0.289
Plummer's adenoma	2 (1.8)	1 (1.6)	0.999
Papillary carcinoma	10 (8.8)	9 (14.3)	0.312
Follicular carcinoma	2 (1.8)	—	0.539
Papillary microcarcinoma	4 (3.5)	3 (4.8)	0.701

*Thyroid function n (%)*			
Hyperfunctioning	32 (31.1)	19 (33.9)	0.725
Normally functioning	56 (54.4)	31 (55.4)	0.999
Underactive	15 (14.6)	6 (10.7)	0.626

Postoperative days, mean ± SD	2.6 ± 0.9	2.9 ± 1.1	0.207^b^

*Operative time, n (%)*	—	—	0.155
1 hour	16 (14.0)	6 (9.5)	—
2 hours	86 (75.4)	45 (71.4)	—
3 hours	12 (10.5)	9 (14.3)	—
>3 hours	—	3 (4.8)	—

^a^Pearson's chi-square test. ^b^Mann–Whitney *U* test.

**Table 3:** Biochemical parameters expressed as median and IQR.

	Normocalcemic	Hypocalcemic	*p* value
TSH, *μ*U.I./ml	1.22 (0.77–2.10)	1.02 (0.49–1.73)	0.081
FT3, pmol/L	5.02 (4.54–5.56)	5.07 (4.60–5.59)	0.802
FT4, pmol/L	16.27 (14.33–18.33)	16.22 (14.35–17.57)	0.744
Vitamin D, ng/mL	28.7 (23.5–40.5)	19.57 (7.10–24.84)	**0.012**
Preoperative serum PTH, pg/mL	44.65 (34.62–68.82)	54.30 (39.18–62.74)	0.799
Albumin, g/L	57.95 (55.40–60.73)	57.80 (55.70–60.45)	0.991
Hgb, g/dL	14.20 (13.20–14.90)	13.50 (12.90–14.63)	0.058

IQR, interquartile range; TSH, thyroid-stimulating hormone; FT3, free triiodothyronine; FT4, free thyroxine; PTH, parathyroid hormone; Hgb, hemoglobin.

**Table 4:** Univariate and multivariate logistic regression analyses to estimate the risk of hypocalcemia.

	Crude OR (95% CI)	*p* value	Adjusted OR (95% CI)	*p* value
Preoperative serum 25-OHD		0.012		0.013
Normal	1		1	
Deficiency	14.82 (1.59–59.70)		14.21 (1.75–115.73)	

Gender		0.216		0.230
Male	1		1	
Female	5.36 (0.95–5.27)		5.00 (0.360–69.60)	

Smoking		0.298		0.321
No	1		1	
Yes	3.75 (0.89–7.85)		3.45 (0.217–54.65)	

Tumor		0.918		0.961
No	1		1	
Yes	1.18 (0.63–3.28)		1.08 (0.05–22.57)	

Obesity		0.057		0.095
No	1		1	
Yes	1.85 (0.98–3.48)		1.65 (0.89–4.95)	

## Data Availability

The data used to support the findings of this study are available from the corresponding author upon request.
